# The Green-Eyed Phantom

**Published:** 1879-12-20

**Authors:** 


### *THE GREEN-EYED PHANTOM.*

Shakspeare never delineated a phase of human passion with more concise and striking words than when he defines jealousy in his peculiar epigrammatic style. Jealousy not only makes the meat it feeds upon, but, ghoul-like, it will exhume every "trifle light as air" buried in the past, and devour it gradually rather than forego a feast upon some kind of the disgusting viands that is craved by its abnormal appetite.

That it is abnormal, is proven by the fact that the food it feeds upon is antagonistic to a healthy passional condition, or to change the metaphor, as there comes in here very *apropos* an illustration embodied in what we might call a back-handed, but striking definition of jealousy that we heard given by a gentleman of our acquaintance not long since. Being a married man of experience, he was one day lecturing a friend, who is also a Benedict, and a very jealous one, consequently always on the lookout for imaginary breakers on the sea of his matrimonial peace. The former, in the course of his lecture to the other about courting misery as some old rheumatic woman nurses her many ailments, concluded his remarks by saying, he never could see the sense of a man *looking for what he did not want to find.*

That told the whole story in a few words. A man under the control of jealousy's baleful passion never desires to have "trifles light as air" "made confirmation strong as proofs of Holy Writ," and at the same time he will leave no effort untried in the search for "what he does not want to find."

That there should be jealousy among women, is no matter of surprise to the student of woman's nature, and her place in creation, and when it goes no farther than to incite a commendable rivalry to excel in all that makes a perfect womanhood, morally, mentally and physically, it is well that it should exist as a part of her emotional organization. Also, it is in accordance with natural laws that there should be frequently heart-burnings and exhibitions of jealousy between men and women—the peasant at his plow, and the milkmaid at her dairy are as often the victims of its pangs as the lovers in homes of wealth.

But the most unworthy phase of this passion is that displayed between men as brothers in one common humanity or co-operators in the same noble work. It is also a cause for surprise that it should exist in these conditions, since, when two or more persons are actuated by the same worthy motives, it seems foreign to the nature of the work itself, that they who are engaged in it should have jealous fears in regard to the success of the other co-workers, or egotistical desires to outstrip them merely for the sake of reaching the goal, and at any cost to the feelings or the failure of others.

It cannot be denied that jealous motives and actions are often instigated by ignoble ambition. A genuine emulation desires to excel for the sake of the excellence in the object of its aspirations, and also wishes others to reach up after the same attainable good and to be benefited by its own beneficence. Opposition and rivalry, born of jealousy, are antagonistic to the legitimate actor in a noble cause, because he is incapable of all passions that will jeopardize the progress of the work, and detract from its dignity and usefulness. He rejoices at the triumphs of the cause for which he labors; he rejoices at the success of all who are zealously and worthily engaged in its prosecution.

Jealousy among men of any profession or avocation is to be deplored. The feeling in these instances is so unworthy, it is the established custom for members of the same profession or trade to refrain from evincing a bitter rivalry towards each other. If they feel it, there is a tacit understanding that to give it expression is a violation of professional etiquette, and an evidence of a great want of proper education and refinement.

There is only one instance where this feeling among confreres in medicine should be cherished, and that is where it is of the kind that will establish an earnest and watchful care of each other's rights, interests and position as brethren in a common cause; where it gives eternal vigilance to the character and attitude of all orthodox theories and supporters in the fraternity, and places a sentinel who will neither slander nor sleep to guard the gate of Medical science from every strange and profane foot that would seek to tread the hallowed ground within. This is the jealousy—not a phantom of the brain, but a vital and ever-present reality, that we should see exhibited by our brethren of the Healing Art—a jealousy that will enhance the dignity and exaltation of the profession—give it character and utility that will ensure its steady progressiveness, incite its still greater possibilities, and call for the benisons of suffering humanity throughout the world. We trust that the past annual cycle of time, with its many events and reminiscences, may take with it all bickering, antagonism, and petty jealousies, and the dawn of the new year usher in a purer and larger growth in the profession of medicine from the Eastern to the Western boundaries of our whole country, especially would we desire the continuance of a generous and helpful attitude in the members of medical journalism and aspirations after a still higher and advanced exposition of medical truths and their scientific application. We who expound these truths should never forget that "the world moves," and we must move with it, being deeply impressed with the great responsibility of our position, the serious and almost sacred trust we assume as expositors of medical lore and teachers of those who desire more light upon the theories and practices of medicine; and, most of all, we, as instructors, should be united in a bond of interest and fraternity that can never be severed by any unworthy motive or action.

To friends and patrons of the RECORD, whose continued patronage and fraternal assistance we so warmly apprreciate, we extend the greetings of a happy New Year. T. S. P.

